# Hypofractionated Irradiation Has Immune Stimulatory Potential and Induces a Timely Restricted Infiltration of Immune Cells in Colon Cancer Tumors

**DOI:** 10.3389/fimmu.2017.00231

**Published:** 2017-03-08

**Authors:** Benjamin Frey, Michael Rückert, Julia Weber, Xaver Mayr, Anja Derer, Michael Lotter, Christoph Bert, Franz Rödel, Rainer Fietkau, Udo S. Gaipl

**Affiliations:** ^1^Department of Radiation Oncology, Universitätsklinikum Erlangen, Friedrich-Alexander-Universität Erlangen-Nürnberg, Erlangen, Germany; ^2^Department of Radiotherapy and Oncology, University Hospital of Frankfurt, Johann Wolfgang-Goethe Universität, Frankfurt am Main, Germany

**Keywords:** hypofractionated radiotherapy, colorectal cancer, tumor-infiltrating immune cells, macrophages, antigen-presenting cells, CD8^+^ T cell, tumor cell-specific IgM, immunogenic radiotherapy

## Abstract

In addition to locally controlling the tumor, hypofractionated radiotherapy (RT) particularly aims to activate immune cells in the RT-modified microenvironment. Therefore, we examined whether hypofractionated RT can activate dendritic cells (DCs), induce immune cell infiltration in tumors, and how the chronology of immune cell migration into tumors occurs to gain knowledge for future definition of radiation breaks and inclusion of immunotherapy. Colorectal cancer treatments offer only limited survival benefit, and immunobiological principles for additional therapies need to be explored with preclinical models. The impact of hypofractionated RT on CT26 colon cancer tumor cell death, migration of DCs toward supernatants (SN) of tumor cells, and activation of DCs by SN were analyzed. The subcutaneous tumor of a BALB/c-CT26 mouse model was locally irradiated with 2 × 5 Gy, the tumor volume was monitored, and the infiltration of immune cells in the tumor was determined by flow cytometry daily. Hypofractionated RT induced a mixture of apoptotic and necrotic CT26 cells, which is known to be in particular immunogenic. DCs that migrated toward SN of CT26 cells particularly upregulated the activation markers CD80 and CD86 when in contact with SN of irradiated tumor cells. After hypofractionated RT, the tumor outgrowth was significantly retarded and in the irradiated tumors an increased infiltration of macrophages (CD11b^high^/F4-80^+^) and DCs (MHC-II^+^), but only between day 5 and 10 after the first irradiation, takes place. While CD4^+^ T cells migrated into non-irradiated and irradiated tumors, CD8^+^ T cells were only found in tumors that had been irradiated and they were highly increased at day 8 after the first irradiation. Myeloid-derived suppressor cells and regulatory T cells show regular turnover in irradiated and non-irradiated tumors. Tumor cell-specific anti-IgM antibodies were enhanced in the serum of animals with irradiated tumors. We conclude that hypofractionated RT suffices to activate DCs and to induce infiltration of innate and adaptive immune cells into solid colorectal tumors. However, the presence of immune cells in the tumor which are beneficial for antitumor immune responses is timely restricted. These findings should be considered when innovative multimodal tumor treatment protocols of distinct RT with immune therapies are designed and clinically implemented.

## Introduction

A promising treatment strategy for solid tumors is the combination of classical tumor therapies namely surgery, radiotherapy (RT), and chemotherapy (CT) with immunotherapy (IT) (1). There is a strong need for rational and well-deliberated approaches of RT–drug combinations on the basis of the molecular understanding of radiobiology and immunology (2–4) since knowledge about the most beneficial time point for radiation breaks and inclusion of IT is scarce.

In high-income countries, more than 50% of cancer patients receive RT as part of their tumor treatment (5). RT induces DNA damage that results in tumor cell cycle arrest and ideally in tumor cell death. The applied amount of radiation is measured in gray (Gy) and aside from the total irradiation dose, the dose fractionation has a substantial impact on therapy outcome. A conventional fractionation scheme comprises 1.8–2.2 Gy per day, five times a week. Although different variations of RT have been clinically evaluated and are now standard options. While hyperfractionated regimens deliver a high number of small treatment doses (0.5–2.2 Gy per day), hypofractionation consists of less fractions with increased doses (3–20 Gy per day) (6) and the latter is considered as being particularly immunogenic (7).

Aside from the effect of RT on DNA, it can also influence immunological responses (8). This can help to fight the tumor locally and at distant, metastasized sites. The regression of tumors distant from the radiation field was named abscopal effect by Mole (9). With the advanced understanding of the immune system’s role in radiation biology, it is hypothesized that such effects are due to a systemic antitumor immune response. One fact among many others who support this hypothesis is that abscopal effects cannot be observed for mice deficient in functional adaptive immune cells (10).

Generally, radiation might change the tumor cell phenotype and/or the tumor microenvironment. Tumor cells increase the surface expression of immunogenic molecules, including adhesion molecules, death receptors, stress-induced ligands, cryptic antigens, and stimulatory molecules, such as MHC-I and CD80, thereby becoming more sensitive to T cell-mediated cytotoxicity. Additionally, in the tumor microenvironment, pro-inflammatory molecules and danger signals increase (11–13). Immune cells are recruited into the tumor and should be stimulated by additional immune modulation (14). Radiation regimens have to be improved and adjusted to maximize immunostimulatory functions for successful combination with other treatments, including IT.

Colorectal cancer is the third most commonly diagnosed malignancy and the fourth leading cause of cancer-related deaths worldwide and forms malignant cells in the tissues of the colon or rectum (15). Extensive efforts to improve the clinical management of patients with colorectal cancer have been made, but approved treatments only offer limited survival benefit. Therefore, alternative therapeutic strategies such as radioimmunotherapy need to be explored with preclinical animal models (16, 17). It has already become evident that the immune infiltrate including type, density, and location of immune cells within human colorectal tumors predict clinical outcome such that individuals with higher infiltrations of T cells have increased survival independent of the disease stage (18).

We investigated the dynamics of immune cell infiltration into colorectal tumors after local hypofractionated irradiation to define optimal time points for additional immune modulations and radiation breaks to protect the infiltrating immune cells. We used the carcinogen-induced murine colon carcinoma CT26 colon adenocarcinoma model for our examinations (19) as responses to immune modulations are similar to those in humans (20).

## Materials and Methods

### Cell Culture

Mouse colon adenocarcinoma cell line CT26.WT (CT26 cells) was cultured in RPMI 1640 (with stabile glutamine) supplemented with 10% fetal bovine serum (FBS), 100 U/ml penicillin, and 100 μg/ml streptomycin (subsequently referred to as R10). CT26 cells tested negatively for mycoplasma contamination were maintained in a 5% CO_2_ atmosphere at 37°C and 95% relative humidity to achieve optimal cell growth. All cell culture methods were performed in laminar flow hoods to avoid microbiological contamination.

### Treatment of CT26 Cells and Cell Death Analyses

The 3 × 10^6^ CT26 cells were seeded in 75 cm^2^ culture flasks, supplied with R10, and after achieving adherence, treated with ionizing radiation with a single dose of 5 Gy (120 kV, 22.7 mA; Isovolt Titan, GE Inspection Technologies, Hürth, Germany). Mock treated CT26 cells served as controls. After 24 h of incubation, the supernatants (SN) were collected, centrifuged (350 *g*, 5 min, room temperature) to remove remaining cells and stored at −80°C. Subsequent adherent cells were washed with PBS and detached with accutase (Sigma-Aldrich, Steinheim, Germany). Afterward, the cells were centrifuged (350 *g*, 5 min, room temperature) and the cell pellet (together with the pellet from the SN centrifugation) was resuspended in R10. For analysis of cell death, 1 × 10^5^ cells were transferred in 400-μl Ringer solution containing 0.2 mg AnxA5-FITC (Life Technologies, GeneArt, Regensburg, Germany) and 0.4 mg PI (Sigma-Aldrich, Munich, Germany). After 30 min incubation at 4°C in the dark, flow cytometry was conducted. Double negative (AnxA5^−^/PI^−^) cells were defined as viable, AnxA5^+^/PI^−^ cells were defined as apoptotic, and double positive (AnxA5^+^/PI^+^) cells were defined as necrotic.

### Colony Formation Assay

CT26 tumor cells were plated in triplicates in 60-mm dishes (Nunc Thermo Fisher, Waltham, MA, USA) at concentrations estimated to yield approximately 100 colonies/dish. Then, the cells were treated with irradiation of 1 × 5 Gy or 2 × 5 Gy. After incubation for approximately 2 weeks, the cells were fixed and adherent cells were stained with methylene blue (Sigma-Aldrich, Munich, Germany) for 30 min. Colonies with >50 cells were scored.

### Generation of Dendritic Cells (DCs) from Mouse Bone Marrow

Generation of DCs from mouse bone marrow was performed according to Lutz et al. (21). At day 0, femurs and tibiae of 8- to 10-week-old female BALB/c mice were removed and purified from surrounding skin and muscle tissue. For disinfection, intact bones were left in 70% ethanol for 5 min and were washed with RPMI 1640 afterward. Subsequently, the articular heads of each bone were cut off and the bone marrow was flushed out. After cell clusters had been disintegrated, the cell suspension was centrifuged (350 *g*, 5 min, room temperature). Then, the cell pellet was resuspended in R10 supplemented with β-mercaptoethanol (0.05mM) and freshly added 200 U/ml mouse GM-CSF (referred to as DC medium). Cells were counted and 2 × 10^6^ bone marrow leukocytes were seeded per 100 mm PS bacteriological Petri dish (Falcon^®^, Corning, NY, USA) containing 10 ml DC medium. At day 3, 10 ml fresh DC medium was added per plate. At days 6 and 8, half of the SN per plate was collected and centrifuged. Thereafter, the cell pellet was resuspended in 10 ml fresh DC medium and returned to the plate. At day 10, DCs were harvested.

### Transwell Migration Assay and Analyses of Activation of DCs

At day 10 of DC cultivation, DCs were harvested, counted, and adjusted to 1.25 × 10^6^ DCs/ml DC medium. SN from the irradiated CT26 cells were thawed on ice and, afterward, 1.5 ml SN per approach was placed in the bottom of a well of a six-well plate (Greiner Bio-One, Frickenhausen, Germany). A cell permeable membrane (with 3.0-μm pore size; Greiner Bio-One, Frickenhausen, Germany) was attached to each well and 800-μl DC cell suspension (containing 1 × 10^6^ cells) was transferred on the upper side of the membrane. The six-well plates were stored in a cell incubator at 37°C overnight (14 h).

For analysis by flow cytometry, migrated cells had to be collected. Therefore, each membrane was carefully lifted with tweezers, and the bottom side was washed with cell suspension of the respective well to collect these cells. Then, the cell suspension was collected from each well and strongly adherent cells were removed by rinsing the well with cold PBS. After centrifugation, each cell pellet was resuspended in Fc block solution [PBS, 10% inactivated FBS, 0.001% Fc-Block, CD16/32 (ebioscience, Frankfurt, Germany)] and incubated for 10 min at 4°C in the dark to prevent non-specific binding of antibodies to Fc receptors.

Cell suspension was distributed to three 1.4 ml PP tubes (Micronic, AR Lelystad, The Netherlands) and antibody solution [MHCII-e450 (0.4 μg/ml, eBioscience, Frankfurt, Germany), CD80-PE (0.4 μg/ml, BD Pharmingen, New York, NY, USA), and CD86-Alexa^®^ Fluor700 (0.4 μg/ml, BD Pharmingen. New York, NY, USA) diluted in FACS buffer (PBS, 2% inactivated FBS)] was added. After incubation for 30 min at 4°C in the dark, cells were washed with FACS buffer and resuspended in it. Further, SN were also directly added to DCs and the expression of the activation markers CD80 and CD86 was analyzed similarly 24 and 48 h afterward. Cells were analyzed by flow cytometry (Gallios, BeckmanCoulter Inc., Krefeld, Germany), and the number of MHCII^+^ cells was defined as the number of migrated DCs. Gating on MHCII^+^ cells was performed for analysis of the mean fluorescence intensity of cells stained with maturation markers CD80 and CD86.

### Animal Studies

The animal studies were approved by the “Regierung von Mittelfranken” and conducted according to the guidelines of the Federation of European Laboratory Animal Science Associations and the “Gesellschaft fuer Versuchstierkunde.” The BALB/c mice (Janvier Labs, Le Genest-Saint-Isle, France) were kept under controlled SPF conditions of humidity (55 ± 5%), temperature (22 ± 2°C), 12/12-h light–dark cycles and received a special diet and water *ad libitum*.

### Injection of CT26 Cells and Measurement of Tumor Growth

Before injection of CT26 cells in BALB/c mice, the colon adenocarcinoma cells were harvested and washed twice with Ringer solution. Thereafter, CT26 cells were counted with the Neubauer-improved counting chamber and percentage of dead cells was determined using trypan blue staining. Concentration was adjusted to 4 × 10^6^ viable CT26 cells/ml Ringer solution. Mice were anesthetized with isoflurane, and injection of 1.2 × 10^6^ CT26 cells in 300-μl Ringer solution was administered subcutaneously in the shaved, disinfected right flank. Tumor width and length were measured using a digital caliper with a measurement accuracy of 0.1 mm and tumor volume was calculated according to the following formula (22): volume (mm^3^) = 0.5 × width^2^ (mm^2^) × length (mm).

### Treatment of CT26 Tumors with RT

At days 8 and 12 after tumor cell injection, local irradiation of the tumor was performed. For this, three mice that had been anesthetized with isoflurane were placed into a purpose-built Plexiglas^®^ (Evonik Industries AG, Darmstadt, Germany) box at a time and inhalation anesthesia was maintained during the whole process to prevent movement of the mice. Tumors were irradiated with a dose of 5 Gy each day using a linear accelerator unit with 6 MV and a focus-skin distance of 1,000 mm. In order to protect healthy tissue, the gantry of the linear accelerator was rotated to 340° as previously described by our group (23).

### Tumor Resection and Blood Samples

For tumor resection, terminal isoflurane anesthesia of mice was applied. At each indicated time point, tumors of three animals were independently analyzed. Blood samples were taken by cardiac puncture and were transferred into heparinized microtainer tubes (BD Microtainer, New York, NY, USA) immediately thereafter. Following centrifugation (12,000 *g*, 10 min, room temperature), to separate serum from cellular components, mice sera were transferred into reaction tubes and stored at −20°C until further usage.

### Tumor Dissociation Procedure

Tumor dissociation was conducted with the mouse tumor dissociation kit (Miltenyi Biotec, Bergisch Gladbach, Germany) according to manufacturer’s instructions with minor modifications. In brief, following removal, tumors were cut into 2–4 mm pieces and transferred immediately into tubes containing the enzymatic mix. Tubes were placed on the gentleMACS™ Dissociator (Miltenyi Biotec, Bergisch Gladbach, Germany) and the predissociation program was run. After incubation for 40 min at 37°C, the final dissociation program was executed. Cell suspension was then pipetted through a 70-μm cell strainer into a 50 ml tube. Subsequent to centrifugation (300 *g*, 7 min, room temperature), the cell pellet was resuspended in RPMI 1640, and cells were counted using the Neubauer improved hemocytometer.

### Measurement of Tumor-Infiltrating Immune Cells

After centrifugation (300 *g*, 7 min, room temperature), cells were resuspended in Fc block buffer and incubated for 10 min at 4°C. Cell suspensions were distributed into 1.4 ml PP tubes and for panel 1 [CD4-FITC (0.5 μg/ml, BD Pharmigen, New York, NY, USA), CD8a-PE (1:500, Miltenyi Biotec, Bergisch Gladbach, Germany), NK 1.1-APC (1:500, Miltenyi Biotec, Bergisch Gladbach, Germany)], panel 2 [CD11b-FITC (0.5 μg/ml, BD Pharmigen, New York, NY, USA), F4/80-Alexa Fluor^®^647 (1:500, Invitrogen, Darmstadt, Germany), LY-6G(GR1)/LY-6C-V450 0.4 μg/ml, BD Horizo, New York, NY, USA], and panel 3 [MHC class II(I-A/I-E)-eFluor^®^450 (0.4 μg/ml, eBioscience, Frankfurt, Germany)], staining solutions were added. After incubation for 30 min at 4°C in the dark, cells were washed with FACS buffer and resuspended in FACS buffer [containing 7-AAD (BioLegend, San Diego, CA, USA, 1:500) for exclusion of necrotic cells]. After staining, infiltrated immune cells were detected using flow cytometry. Gating was performed on 7-AAD negative (non-necrotic) cells. The percentage of positive cells was determined for each cell marker or for combinations of various markers. Detection of regulatory T cells (Tregs) (panel 4) was performed as follows: cell suspension was incubated with CD4-VioBlue^®^ (1:40, Miltenyi Biotec, Bergisch Gladbach, Germany) and CD25-Alexa Fluor^®^488 (2.5 μg/ml; eBioscience, Frankfurt, Germany) for 10 min at 4°C in the dark. Thereafter, 500 μl FACS buffer (PBS containing 2% FCS) was added and cells were centrifuged (350 *g*, 5 min, 4°C). The cell pellet was resuspended in fixation/permeabilization solution and incubated for 30 min at 4°C in the dark. Cells were washed with FACS buffer and then with permeabilization solution. Afterward, cells were resuspended in permeabilization buffer. Following incubation for 5 min at 4°C, FoxP3-APC antibody (1:40, Miltenyi Biotec, Bergisch Gladbach, Germany) was added and incubated for 30 min at 4°C in the dark. Finally, cells were washed with permeabilization buffer and the cell pellet was resuspended in FACS buffer.

### Analysis of Tumor Cell-Specific IgM Antibodies in Sera of CT26 Colon Tumor-Bearing Mice

For determination of tumor cell-specific IgM antibodies, indirect immunofluorescence analysis was used. Mice sera were thawed on ice and 1 μl of the respective serum sample was co-incubated with 1 × 10^5^ viable CT26 cells for 1 h at 4°C. Thereafter, cells were washed with PBS/10% FBS. The amount of bound antibodies was analyzed by adding staining solution [5.8 μg/ml FITC-conjugated goat anti-mouse IgM (Invitrogen, Darmstadt, Germany)] for 1 h at 4°C in the dark. After washing, cells were resuspended in PBS/10% FBS. Using flow cytometry, the mean fluorescence intensity of CT26 cells per sample was analyzed and equated with the tumor cell-specific IgM antibody level in the serum.

### Flow Cytometry

For cell death analysis, analysis of migrated cells in the transwell migration assays, detection of IgM antibodies, and for investigation of immune cell infiltration in CT26 colon tumors, flow cytometry using GalliosTM and Epics XL MCL was conducted. Both flow cytometers were equipped with a multi-carousel loader unit that made it possible to analyze up to 32 samples automatically in a row. Coulter^®^ Isoton^®^ II diluent functioned as sheath fluid in all experiments. Flow cytometry data were acquired as LMD files, which were analyzed using Kaluza 1.2 software.

## Results

### Hypofractionated RT Reduces Colony Formation and Generates Apoptotic and Necrotic CT26 Tumor Cells

**Figure 1:**
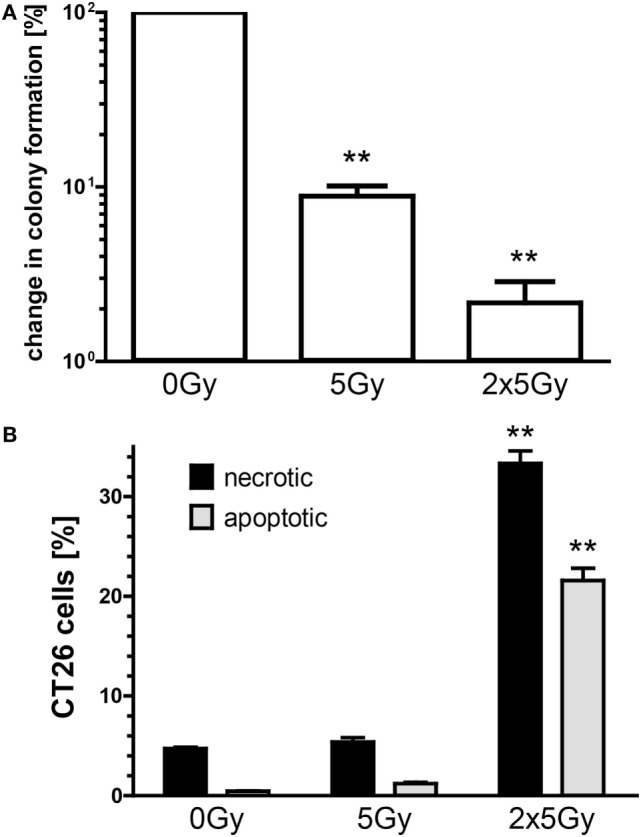
**Hypofractionated irradiation reduces the colony formation and induces apoptosis and necrosis of CT26 cells**. The colony formation was determined by standard colony formation assay **(A)**. After incubation for approximately 2 weeks, the cells were fixed and colonies with >50 cells were scored. The cell death analyses were performed 24 h after single or double irradiation of CT26 colorectal tumor cells with 5 Gy. Cell death was determined by flow cytometry; apoptotic cells (gray) are defined as AxV^+^/PI^−^ cells and necrotic (black) as AxV^+^/PI^+^ cells **(B)**. Joint data of three independent experiments, each performed in duplicates, are presented as mean ± SEM and analyzed by Student’s *t*-test; **p < 0.01.

We first tested *in vitro* whether irradiation with a single dose of 5 Gy and repeated irradiation with 2 × 5 Gy (hypofractionated RT) succeeds to reduce the colony formation of colorectal cancer cells and also induces immunogenic cell death forms. Both a single irradiation dose with 5 Gy and a hypofractionated irradiation dose significantly reduced the colony formation of CT26 cells (Figure 1A). However, a second irradiation dose of 5 Gy is needed to significantly increase the percentage of apoptotic and necrotic tumor cells as early as 1 day after treatment (Figure 1B).

### SN of Tumor Cells Induce Migration and SN of Irradiated Tumor Cells Increase Activation of DCs *In Vitro*

**Figure 2:**
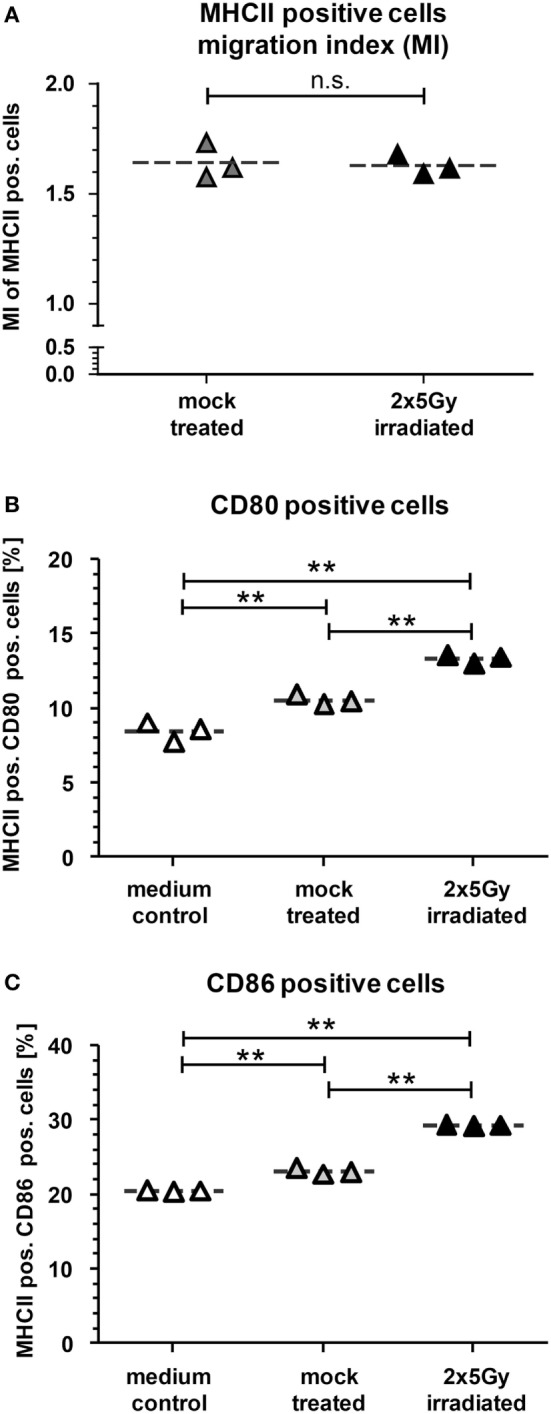
**Dendritic cells (DCs) migrate toward supernatants (SN) of CT26 cells and are particularly activated by SN of irradiated CT26 cells**. Bone marrow-derived DCs from BALB/c mice (mDCs) were harvested and seeded to the upper chamber of a transmigration system (3.0 μm pore size). The lower chamber was filled with cell culture SN obtained from CT26 tumor cells 24 h after irradiation with 2 × 5 Gy on consecutive days or with SN of mock-treated cells. After 14 h of incubation at 37°C, the transmigration index (MI), reflecting the migration of mDCs toward SN of the tumor cells versus the medium only control, was determined **(A)** and the expression of CD80 **(B)** and CD86 **(C)** on the MHCII^+^ transmigrated cells was determined by flow cytometry. Joint data of three independent experiments are presented as mean ± SEM and analyzed by Student’s *t*-test; **p < 0.01.

**Figure 3:**
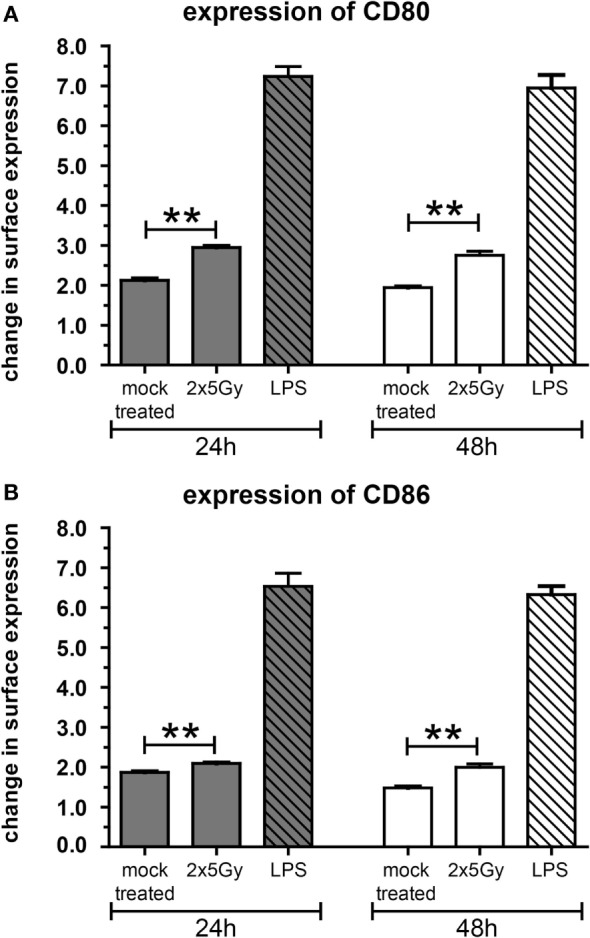
**The activation of dendritic cells (DCs) by supernatants (SN) of irradiated CT26 cells is independent of the migration**. Bone marrow-derived DCs from BALB/c mice (mDCs) were incubated at 37°C in SN obtained from CT26 tumor cells 24 h after irradiation with 2 × 5 Gy on consecutive days, in SN of non-irradiated mock treated CT26 cells, or in medium containing lipopolysaccharide (LPS). The expression of CD80 **(A)** and CD86 **(B)** on mDCs was analyzed after 24 and 48 h *via* flow cytometry. Representative data of one out of three independent experiments each performed in triplicates are presented as mean ± SEM and analyzed by Student’s *t*-test; **p < 0.01.

To further characterize the immunostimulatory potential of the irradiated tumor cells, a transwell migration assay was performed with murine DCs (mDCs) (Figure 2). The transmigration as well as the activation status of the migrated DCs was analyzed. SN of tumor cells attracted mDCs resulting in over 1.5× more cells migrating through the insert compared to the medium control. However, this was independent of whether the cells were irradiated or not (Figure 2A). However, only SN of the irradiated tumor cells induced a significant higher increase in the percentage of migrated mDCs showing enhanced expression of the activation markers CD80 and CD86 compared to mock treated and medium controls (Figures 2B,C). To test whether mDCs are activated through the process of (trans)migration or by the SN *per se*, mDCs were also directly incubated with SN of mock treated and irradiated tumor cells, respectively. As shown in Figure 3, SN of irradiated CT26 cells induced a significant increased expression of the activation markers CD80 and CD86 on mDCs compared to SN of the mock treated control. This was observed 24 and 48 h after incubation with the SN (Figures 3A,B). However, the increased expression of CD80 and C86 on mDCS induced by SN of irradiated CT26 cells was weaker compared to that induced by lipopolysaccharide (Figure 3).

### Local Tumor Control of CT26 Tumors in BALB/c Mice Can Be Achieved with 2 × 5 Gy Hypofractionated Irradiation

**Figure 4:**
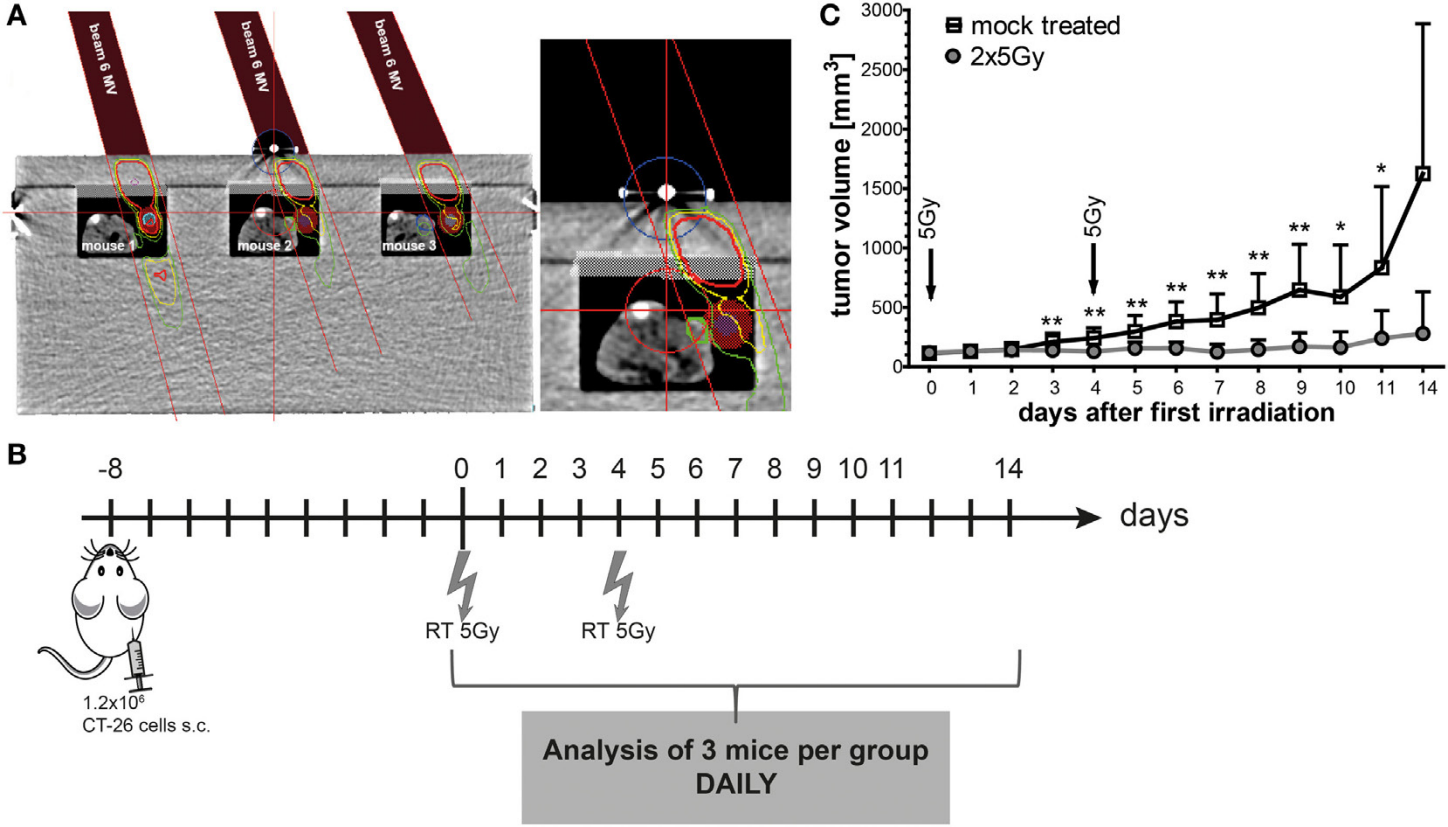
**Hypofractionated radiotherapy (RT) results in local control of CT26 colon cancer tumors in BALB/c mice**. The planning of the irradiation was conducted using a computer tomography image of the irradiation box and tumor-bearing mice with Philips pinnacle software to obtain an optimal target volume. Afterward, the dosimetry of the irradiation was performed manually with a calibrated ionization chamber. To further protect the normal tissue, the gantry of the 6-MV linear accelerator was rotated to 340°. Tumors of three anesthetized mice can be irradiated locally at once and the dose distribution (colored areas) shows that only the tumor and not the rest of the mouse is exposed to radiation **(A)**. The tumor volumes were determined daily. Up to day 4 after the first irradiation with 5 Gy, the infiltration of immune cells in the tumors was monitored in tumors of three mice from each group **(B)**. Hypofractionated irradiation with 2 × 5 Gy resulted in good tumor control **(C)**; *p < 0.05, **p < 0.01; *n*: variable: at the starting point *n* = 40, with three mice less each following day per treatment group.

To irradiate the tumor-bearing BALB/c mice, we manufactured a Plexiglas^®^ box, which allows the irradiation of three mice at once (Figure 4A). The tumors were locally irradiated (colored dose distribution area; Figure 4A) and treatment planning was conducted using a computer tomography image of the Plexiglas irradiation box and tumor-bearing mice with Philips pinnacle software (Best, Netherlands). To protect normal tissue (body of the mouse 1, 2, and 3), the gantry of the linear accelerator was rotated to 340° and the tumor area was then irradiated with a dose of 5 Gy with 6-MV photons and a focus-skin distance of 1,000 mm. The mice were anesthetized before placing them in the box and during the whole irradiation procedure. On day 8 after the injection of CT26 tumor cells in BALB/c mice, the mice were irradiated with 2 × 5 Gy in a 4-day interval. Beginning with the day of the first irradiation, the tumor volume was measured daily for 14 days (Figure 4B). The treatment of tumor-bearing mice with hypofractionated RT delayed the tumor growth significantly and resulted in good local tumor control (Figure 4C).

### Infiltration of Immune Cells into the Irradiated Tumor Occurs in a Narrow Time Frame

**Figure 5:**
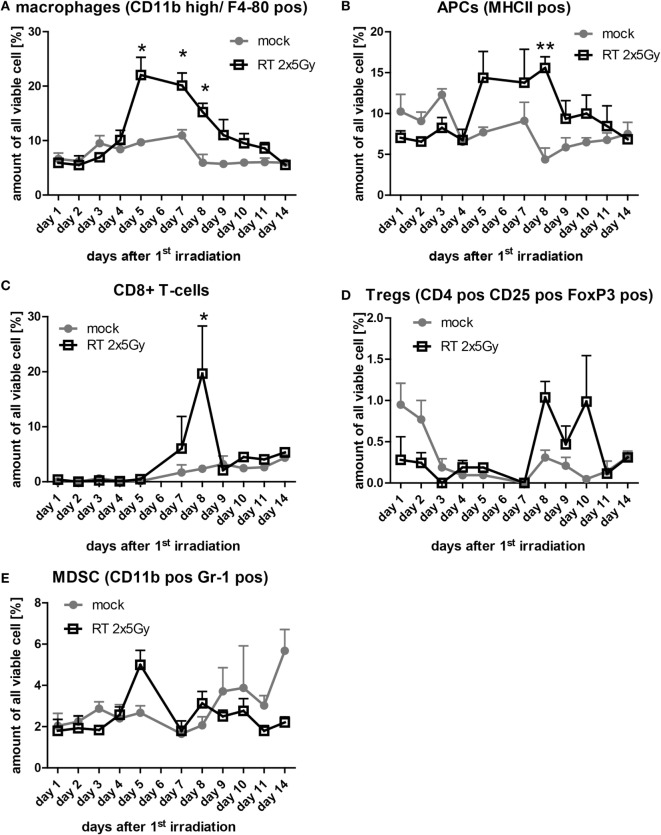
**The infiltration of immune cells in irradiated tumors is timely restricted**. At each day of the examination period, three tumors of each group were separately enzymatically dissociated and consecutively analyzed for immune cell infiltration by flow cytometry. The amount of the indicated immune cells out of all analyzed viable cells is displayed **(A–E)**. Data of three independent tumors are presented as mean ± SEM and analyzed by Student’s *t*-test; *p < 0.05, **p < 0.01.

Next, we were interested whether hypofractionated irradiation induces immune cell infiltration into the tumor and, in particular, the chronology of this process. Each day of the observation period three mice per group were sacrificed for the analysis of tumor-infiltrating leukocytes. Elevated numbers of tumor-infiltrating macrophages (CD11b high/F4-80^+^) and antigen-presenting cells (MHC-II^+^) between day 5 and 10 after the first irradiation were observed in tumors of irradiated mice compared to mock treated tumors (Figures 5A,B). The amount of CD8^+^ T cells in irradiated tumors did not differ from that of mock-treated tumors, except at day 8, where significantly more cytotoxic T cells were present in irradiated tumors (Figure 5C). CD4^+^ T cells migrated into non-irradiated and irradiated tumors in a similar manner (data not shown). The percentage of Treg (CD4^+^/CD25^+^/FoxP3^+^) in the tumor was low and irradiation with 2 × 5 Gy induced no higher amounts of Treg when compared to the normal turnover in non-irradiated tumors (Figure 5D). The same was observed for myeloid-derived suppressor cells, defined as CD11b^+^/Gr-1^+^ cells (Figure 5E). Starting at day 9 after the first irradiation, the amount of immune cells did not differ any more between irradiated compared to mock-treated tumors (Figure 5).

### Hypofractionated Irradiation Induces Tumor Cell-Specific IgM Antibodies

**Figure 6:**
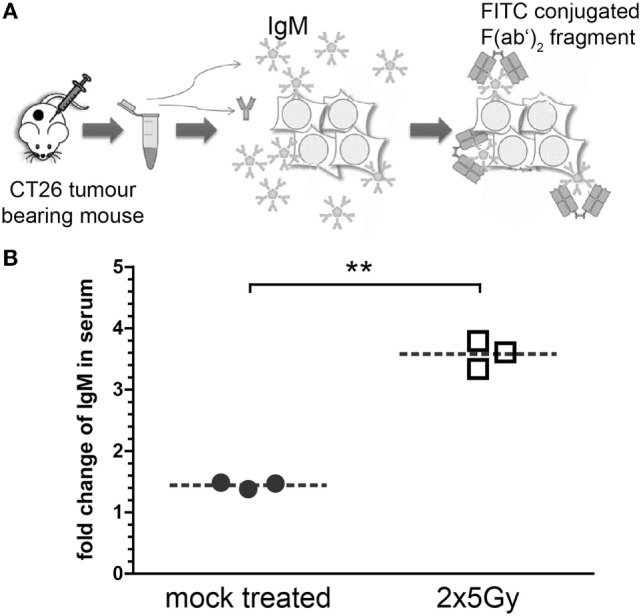
**Hypofractionated irradiation of colorectal tumors induces tumor-cell-specific IgM antibodies**. The sera of the mice whose tumor had been irradiated with 2 × 5 Gy were analyzed for tumor cell-specific IgM antibodies. For this, serum was collected from blood samples taken at the last day of the observation period (see Figure 3). These sera were then co-incubated with viable CT26 cells. IgM antibodies bound to the tumor cells were stained with FITC-conjugated anti-mouse IgM F(ab’)_2_ fragments and analyzed *via* flow cytometry **(A)**. Data of three independent tumor-bearing mice are presented as mean ± SEM **(B)** and analyzed by Student’s *t*-test; **p < 0.01.

To test whether irradiation also affects humoral immune response, tumor cell-specific IgM antibodies were analyzed. For this, blood samples of tumor-bearing mice were taken and the gained serum was co-incubated with CT26 tumor cells. The amount of bound antibodies was analyzed by adding FITC-conjugated goat anti-mouse IgM F(ab’)_2_ fragments (Figure 6A). The analyses by flow cytometry showed that the titer of tumor cell-specific IgM antibodies was significantly higher compared to mock-treated animals only in serum of mice whose tumor had been irradiated (Figure 6B).

## Discussion

Neoadjuvant chemoradiation has been shown to alter the *in situ* immune cell population in rectal cancer. A high CD8^+^ T cell density in the stroma after RCT was associated with a favorable clinical outcome (24). In colorectal cancer, the density of infiltration of lymphocytes is associated with better overall survival and the immune status has emerged as a beneficial tool to improve the management of patients (25). Immunological biomarkers are, therefore, being used more frequently as a tool for the prediction of prognosis and response to therapy in addition to traditional tumor staging (26). However, it is important to consider the spatiotemporal dynamics of different immune cell types that infiltrate into tumors (27).

Currently, several combinations of RT with IT, such as monoclonal antibodies blocking immune checkpoints are being tested in clinical trials, since it is still unknown how to bring these treatment modalities together chronologically to achieve the most beneficial outcome for the patient (28). As a prerequisite to coordinate both treatments, it is mandatory to know the RT-induced immune profile, which can be boosted and harnessed by IT. Therefore, we investigated the infiltration of immune cells into irradiated colorectal cancer tumors (Figure 5).

Hypofractionated irradiation with 2 × 5 Gy induced a significant increased infiltration of cells of the innate immune compartment. Enhanced APCs (macrophages and MHC class II positive cells referred to as DCs) as early as 1 day after the last irradiation were observed. Of note is that the amount of APCs was increased in the CT26 colorectal cancer tumor only after about 3 days.

Our *in vitro* experiments revealed that irradiation of the colorectal tumor cells with 2 × 5 Gy results in a mixture of apoptotic and necrotic tumor cells and in recruitment and activation of DCs (Figures 1–3). Danger signals released by tumor cells might be central for the recruitment of myeloid cells in the tumor (29). While DCs did migrate *in vitro* similarly toward SN of mock treated and irradiated tumor cells, in particular SN of irradiated tumor cells induced an increased expression of the activation markers CD80 and CD86 on DCs. One could speculate that low amounts of danger signals being present under tumor cell culture conditions suffice to recruit DCs and that higher amounts of them being present after irradiation are mandatory to induce an increased expression of activation markers on DCs. High amounts of the danger signal Hsp70 in the extracellular milieu have already been demonstrated to induce an increased expression of CD80 and CCR7 on DCs (30).

*In vivo*, when the APCs dropped again, CD8^+^ T cells were enhanced in the tumor, but stayed there only for around 1 day (Figure 5). This might indicate that the cytotoxic T cells were recruited by the activated APCs. Klug and colleagues have previously demonstrated that gamma irradiation causes normalization of aberrant vasculature in tumors and fosters infiltration of immune cells. This was dependent on reprogramming of macrophages (31). Since normalization of the tumor vasculature seems to be a key factor for enhanced immune cell infiltration, these effects can only be observed *in vivo* and not with *in vitro* model systems. We also did not observe any differences in the migration index of DCs toward SN of non-irradiated compared to SN of irradiated CT26 cells in our *in vitro* migration assay (Figure 2).

Recently, it was shown that hypofractionated irradiation of B16 melanoma tumors with 2 × 12 Gy on consecutive days induced a high infiltration of CD8^+^ T cells at day 5 after the last irradiation. Later on, the amount of the cytotoxic T cells dropped again (32). Our data also reveal that CD8^+^ T cells do migrate in solid tumors that have been irradiated with a hypofractionated protocol. It must be emphasized that the immune cell infiltration takes place in a narrow time window (Figure 5). This knowledge is indispensable for designing strategies for inclusion of additional IT to classical tumor therapies, namely RT, CT, or RCT.

It has become clear that RT and RCT do have the potential to change the tumor and its microenvironment (33) and that radiation exposure is reflected locally and systemically (34). Innovative IT approaches should consider the dynamics of radiation-induced immune cell infiltration into tumors since the immune cells should be activated in the modified environment. Further, hypofractionated radiation might be of advantage in radioimmunotherapy since wide intervals between the single irradiations do exist that might allow the immune cells to act and react (35). In particular, cytotoxic T cells and B cells do have a radiation sensitive phenotype and might be affected when being present in the tumor during re-irradiation (36). The infiltration of immune suppressive cells such as Treg and MDSC was not significantly influenced by hypofractionated RT (Figure 5). However, a slight increase of Treg was seen at days 8–10 after the first irradiation and, therefore, mainly following the infiltration of CD8^+^ T cells. An optimal re-irradiation of the tumor would in this case be at day 9–10 where the cytotoxic T cells have already left and immune suppressive Treg cells are still inside the tumor.

While in many cases it has been demonstrated that the cellular component of the adaptive immune system and, in particular, CD8^+^ T cells is key for radioimmunotherapy-induced antitumor immune responses, much less is known about the humoral part (37). We found tumor cell-specific IgM antibodies to be enhanced in the serum of mice whose tumors had been irradiated with 2 × 5 Gy (Figure 6). Splenocytes of mice whose renal cancer tumor was treated with radioimmunotherapy secreted higher amounts of tumor cell-specific IgM antibodies, indicating that a systemic antitumor immune response was triggered (38). We show for the first time that hypofractionated RT *per se* might be sufficient to provoke such humoral antitumor responses. However, the latter are not necessarily involved in abscopal radiation responses, as it has recently been demonstrated with the 67NR mammary carcinoma model and hypofractionated irradiation with 3 × 8 Gy. However, increased IgM was also observed in the irradiated primary tumor (39).

We conclude that hypofractionated RT *in vivo* attracts immune cells into colorectal cancer tumors and is capable of inducing a tumor cell microenvironment that activates DCs. The infiltration of the immune cells is dynamic and, therefore, timely restricted. Cytotoxic CD8^+^ T cells follow the APCs. This knowledge is valuable for designing multimodal radioimmunotherapies: at days of high infiltration of immune cells being involved in antitumor immune responses, RT should be paused and IT should be applied. Consequently, at days of low infiltration of these immune cells and high infiltration of immune suppressive cells, re-irradiation without IT should be performed. Knowledge of how immune cells in the periphery correlate with the observed processes in the tumor will further facilitate the optimization of multimodal radioimmunotherapies (40). The potential synergies of RCT with IT should be exploited to improve the clinical outcome for each patient (41), and the preclinical data presented here on the chronology of immune cell infiltration into tumors after local irradiation should help to optimize of clinical radioimmunotherapy protocols.

## Author Contributions

BF: performed most of the practical work together with JW and MR and drafted the manuscript together with UG. MR: performed the practical work together with BF and JW and wrote the manuscript together with UG and BF. JW: performed the practical work together with BF and MR. XM: performed the *in vitro* DC assays. AD: contributed to the evaluation of the data and the writing of the manuscript. ML: performed the treatment planning for the mouse irradiation. CB: contributed to the design of the work and to the planning of the mouse irradiation protocol. FR and RF: contributed to the design of the work. UG: drafted and designed the study, drafted the manuscript, and wrote it together with BF and MR.

## Conflict of Interest Statement

The authors declare that the research was conducted in the absence of any commercial or financial relationships that could be construed as a potential conflict of interest.
